# Enzyme-Responsive Amphiphilic Peptide Nanoparticles for Biocompatible and Efficient Drug Delivery

**DOI:** 10.3390/pharmaceutics14010143

**Published:** 2022-01-07

**Authors:** Su Jeong Song, Joon Sig Choi

**Affiliations:** 1 Korean Medicine Convergence Research Division, Korea Institute of Oriental Medicine, Daejeon 34054, Korea; song.sj87@kiom.re.kr; 2 Department of Biochemistry, Chungnam National University, Daejeon 34134, Korea

**Keywords:** peptide nanoparticle, doxorubicin, cytotoxicity, drug delivery systems

## Abstract

Self-assembled peptide nanostructures recently have gained much attention as drug delivery systems. As biomolecules, peptides have enhanced biocompatibility and biodegradability compared to polymer-based carriers. We introduce a peptide nanoparticle system containing arginine, histidine, and an enzyme-responsive core of repeating GLFG oligopeptides. GLFG oligopeptides exhibit specific sensitivity towards the enzyme cathepsin B that helps effective controlled release of cargo molecules in the cytoplasm. Arginine can induce cell penetration, and histidine facilitates lysosomal escape by its buffering capacity. Herein, we propose an enzyme-responsive amphiphilic peptide delivery system (Arg-His-(Gly-Phe-Lue-Gly)_3_, RH-(GFLG)_3_). The self-assembled RH-(GFLG)_3_ globular nanoparticle structure exhibited a positive charge and formulation stability for 35 days. Nile Red-tagged RH-(GFLG)_3_ nanoparticles showed good cellular uptake compared to the non-enzyme-responsive control groups with d-form peptides (LD (^L^RH-^D^(GFLG)_3_), DL (^D^RH-L(GFLG)_3_), and DD (^D^RH-^D^(GFLG)_3_). The RH-(GFLG)_3_ nanoparticles showed negligible cytotoxicity in HeLa cells and human RBCs. To determine the drug delivery efficacy, we introduced the anticancer drug doxorubicin (Dox) in the RH-(GFLG)_3_ nanoparticle system. LL-Dox exhibited formulation stability, maintaining the physical properties of the nanostructure, as well as a robust anticancer effect in HeLa cells compared to DD-Dox. These results indicate that the enzyme-sensitive RH-(GFLG)_3_ peptide nanoparticles are promising candidates as drug delivery carriers for biomedical applications.

## 1. Introduction

Peptides are currently being investigated as carriers in delivery systems due to their excellent biocompatibility and biodegradability [1,2,3]. In addition, peptides are involved in important biological mechanisms, which can impart strategic and efficient roles in peptide-based systems [4,5]. For example, cell-penetrating peptides (CPPs) are applied to enhance cellular uptake [6,7]. Positively charged lysine and arginine can induce interactions with cell membranes and deliver cargo molecules such as nanoparticles, small molecules, and genes into the cell. The nuclear localization sequence (NLS) can be used to import proteins into the cell nucleus by nuclear transport, similarly to the gene delivery system that requires penetration into the nuclear membrane for effective gene expression [8,9]. Therefore, NLS has been used as a good strategy for gene delivery. Mitochondrial targeting peptides have also been reported as useful tools for strategic delivery systems [10,11]. Peptides have been researched as valuable biomaterials for novel delivery systems.

Self-assembled nanostructures of amphiphilic peptides (APs) have attracted much attention in drug delivery systems due to their capability in self-assembling induced by their corresponding amino acid structure [12,13,14]. In recent studies, modification of APs has been reported for biomedical applications and formulation mechanisms [15,16]. APs based on a coiled-coil helix bundle have been shown to form hierarchical assemblies [17]. Polypeptides derived from calcium channel segment have been evaluated for application in drug delivery systems [18]. APs containing arginine and valine have been reported as for siRNA delivery systems [19]. Nanostructures based on APs have been reported to possess self-adjuvant properties [20]. Branched amphiphilic peptide capsules have been studied for cellular uptake and retention of encapsulated solutes [20]. Moreover, in a study using molecular dynamics simulation, the encapsulation process and release behavior of an alpha-helical antimicrobial peptide was evaluated [21]. Thus, APs are being actively studied in basic formulations and biomedical applications.

Drug delivery system (DDS) with APs have exhibited potential as biocompatible and efficient delivery carriers. Most therapeutic drugs are hydrophobic and have low solubility in aqueous solutions, which reduces their therapeutic efficacy [22]. Therefore, it is necessary to improve the solubility of the drug via encapsulation [23,24]. APs containing a hydrophilic head and hydrophobic core can encapsulate hydrophobic drugs, which can further improve their therapeutic efficacy [25]. The peptides have great biocompatibility and biodegradability, which are advantageous in DDS [26]. Delivery systems based on polymers have been reported to have significant cytotoxicity because of their low degradability and excessive intracellular accumulation [27]. However, DDS with APs have not yet achieved satisfactory results compared to polymer-based DDS. Therefore, to enhance the efficiency of AP delivery systems, the control and stability of drug release must be improved.

**Figure 1:**
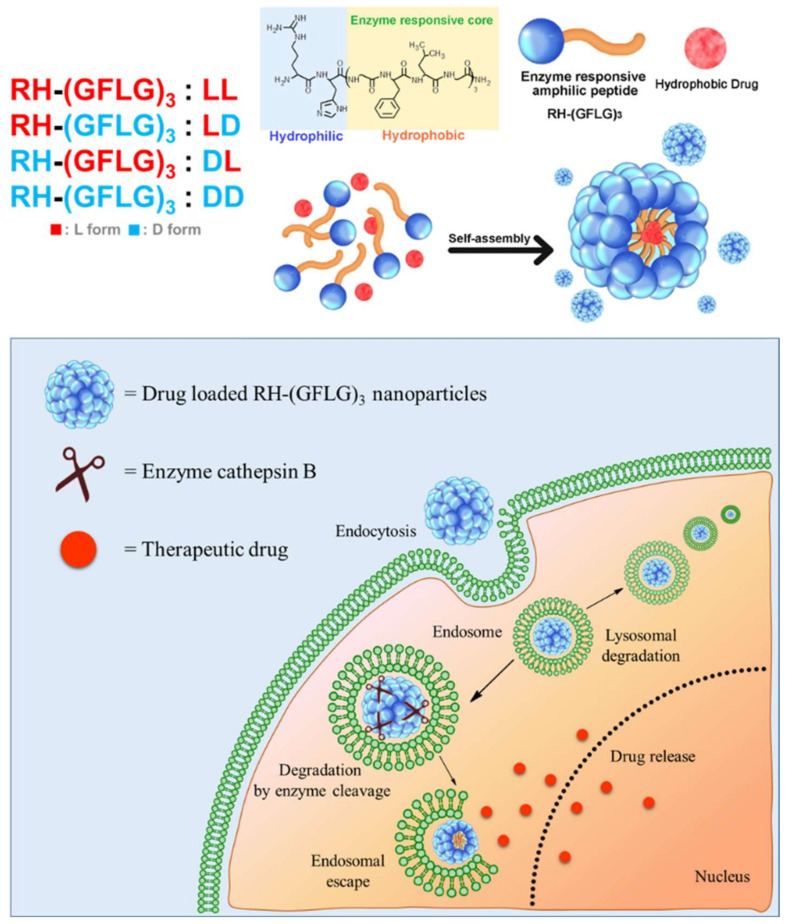
Schematic illustration of RH-(GFLG)_3_ nanoparticles as a drug carrier.

In our previous study, we reported amphiphilic peptide nanorod (Arg-His_3_-Phe_8_, RH_3_F_8_) for biocompatible drug delivery [28]. The amphiphilic peptide nanorod showed formulation stability and improved drug delivery efficiency both in vitro and in vivo. Here, we report an advanced amphiphilic peptide delivery system with modified enzyme-responsive ability for efficient drug delivery (Arg-His-(Gly-Phe-Lue-Gly)_3_, RH-(GFLG)_3_) (Figure 1). Controlled release by stimulative response is a potential strategy for effective drug delivery [29,30]. The Gly-Phe-Leu-Gly (GFLG) oligopeptide can be degraded by the endo/lysosomal enzyme cathepsin B [31,32]. Cathepsin B belongs to a family of endo/lysosomal proteases and plays a vital role in intracellular proteolysis [33]. In addition, it is upregulated in invasive and metastatic cancer cells, which has attracted much attention in efficient and targeted cancer therapy [34,35]. The GLFG peptide containing the hydrophobic peptide leucine, and phenylalanine is introduced as the core of the peptide nanoparticle for effective cancer therapy. The peptide core is anticipated to release the drug at the intracellular level and deliver it specifically to cancer cells. In addition, the nanoparticle structure has a hydrophilic region that contains arginine and histidine. Owing to its positive charge, arginine as a cell-penetrating peptide (CPP) increases cellular uptake [36]. Histidine with an imidazole ring induces endo/lysosomal escape owing to its proton buffering capacity [37]. Moreover, the GLFG core contains phenylalanine and leucine, which is useful in encapsulating hydrophobic drugs [38]. In our preliminary study, we designed three types of peptides, RH-(GFLG)_1_, RH-(GFLG)_2_, and RH-(GFLG)_3_, to identify the optimal number of GFLG peptides for the formation of nanoparticles. The self-assembled RH-(GFLG)_3_ nanoparticles present a globular nanostructure, whereas RH-(GFLG)_1_ and RH-(GFLG)_2_ do not self-assemble because of the lack of hydrophobicity. The repeating GLFG oligopeptides help form the self-assembled structure. The prepared RH-(GFLG)_3_ nanoparticles were evaluated as drug carriers for cancer therapy against a non-enzyme-responsive control group containing D-form peptides (LD (^L^RH-^D^(GFLG)_3_), DL (^D^RH-L(GFLG)_3_), and DD (^D^RH-^D^(GFLG)_3_). Physical characterization of the RH-(GFLG)_3_ nanoparticles was performed using dynamic light scattering (DLS), field emission scanning electron microscopy (FE-SEM), and circular dichroism (CD) spectroscopy. Cellular uptake was examined by confocal microscopy and fluorescence-activated cell sorting (FACS). The cytotoxicity of RH-(GFLG)_3_ was analyzed by 3-(4,5-dimethylthiazol-2-yl)-2,5-diphenyltetrazolium bromide (MTT), lactate dehydrogenase (LDH), and hemolysis assays. Doxorubicin was employed to confirm the drug delivery efficiency. To investigate the enzyme-sensitive ability of RH-(GFLG)_3_, we employed matrix assisted laser mass spectroscopy (MALDI-MS) and an in vitro drug release test. The cellular uptake of doxorubicin was analyzed using confocal microscopy, and the anticancer effect of doxorubicin-loaded RH-(GLFG)_3_ nanoparticles was analyzed both in vitro and in vivo.

## 2. Materials and Methods

### 2.1. Materials

Nile Red, Tween-20, agarose, branched 25 kDa poly-ethylenimine (*b*-PEI), doxorubicin, thiazolyl blue tetrazolium bromide (MTT), cathepsin B enzyme, and tricaine mesylate were purchased from Sigma-Aldrich (Seoul, Korea). Fetal bovine serum (FBS), Dulbecco’s modified Eagle medium (DMEM), 100× antibiotic-antimycotic agent, and Dulbecco’s phosphate-buffered saline (DPBS) were purchased from Gibco Laboratories (Gaithersburg, MD, USA). Regenerated cellulose dialysis membrane (MWCO, 1000 Da, Spectra/Por) was obtained from Spectrum Laboratories (Rancho Dominguez, CA, USA). Hoechst 33342 was purchased from Invitrogen Corp. (Carlsbad, CA, USA). Triton X-100 was purchased from Shinyo Pure Chemicals Co., Ltd. (Osaka, Japan). Cell tracker green CMFDA dye was purchased from Thermo Fisher Scientific (Waltham, MA, USA).

### 2.2. Preparation of Peptide Nanoparticles

RH-(GFLG)_1_, RH-(GFLG)_2_, ^L^RH-^L^(GFLG)_3_ (LL) ^L^RH-^D^(GFLG)_3_ (LD), ^D^RH-^L^(GFLG)_3_ (DL), and ^D^RH-^D^(GFLG)_3_ (DD) were purchased from Peptron Co., Ltd. (Daejeon, Korea). The D-form LD, DL, and DD peptides were introduced as control groups. The peptide nanoparticles were formulated using the sonication method. Briefly, the peptides were dissolved in aqueous solutions to final concentrations of 1–5 mg/mL. The peptide solutions were sonicated for 15 min and stored overnight at 4 °C. Nile Red-tagged peptide nanoparticles were prepared for cellular uptake assays. Nile Red was dissolved in methanol to a concentration of 100 nM, and dried using a nitrogen gas steam. Further, the peptide solution was added to the Nile Red glass containing the Nile Red solution at a molar ratio of Nile Red:peptide = 1:10^5^ and was sonicated for 15 min. After sonication, non-loaded Nile Red was removed using size exclusion spin columns, and the prepared samples were stored at 4 °C.

### 2.3. Physical Characterization

The size of the peptide nanoparticles at concentrations of 0.5 mg/mL was analyzed by dynamic laser light scattering (DLS) with ELS-Z2 (Photal, Otsuka Electronics Co., Ltd., Otsuka, Japan) and a Nano-ZS (Malvern Instruments Ltd., London, UK). The ξ-potential of the peptide nanoparticles was measured using a Nano-ZS. To confirm their stability, the LL and DD nanoparticles were monitored using a DLS instrument every 5 days for 30 days. The morphology of the LL peptide nanoparticles was measured using a field emission scanning electron microscope (FE-SEM). Thereafter, the dried samples of peptide nanoparticles were coated with osmium and analyzed by FE-SEM (Hitachi, Tokyo, Japan) under a 5–10 kV acceleration voltage. The self-assembled structure of the peptide nanoparticles was analyzed using CD spectroscopy (J-1000, Jasco, Hachioji, Japan). The LL and DD peptide nanoparticle solutions were measured in a quartz cuvette at wavelengths of 190 to 260 nm.

### 2.4. Determination of Critical Aggregation Concentration

The critical aggregation concentration (CAC) value of the RH-(GFLG)_3_ nanoparticles was analyzed using the hydrophobic probe Nile Red [39]. Briefly, the peptide nanoparticles were prepared at various concentrations ranging from 0.001 to 0.3 mg/mL to make a final volume of 1 mL. Then, the prepared nanoparticle solutions were mixed with 5 μL of 40 μM Nile Red solution in ethanol to a final concentration of 0.2 μM. The samples were dried to remove the ethanol using nitrogen gas stream and incubated for 1 h at room temperature (25 °C). After incubation, prepared samples were measured using fluorescence spectroscopy (PerkinElmer, Waltham, MA, USA) at an excitation wavelength of 515 nm and emission wavelengths of 550–750 nm. The CAC of the peptide nanoparticles was analyzed by fluorescence intensity at 650 nm.

### 2.5. Cell Culture

HeLa (cervical cancer, Korea Cell Line Bank, Seoul, Korea) and SW480 (colon cancer, Korea Cell Line Bank) cells were incubated in cell media containing DMEM 89%, FBS 10%, and 0.1 mg/mL of an antibiotic-antimycotic agent. The cells were incubated in a cell incubator (37 °C, 5% CO_2_).

### 2.6. Cytotoxicity of Peptide Nanoparticles

The cytotoxicity of the RH-(GFLG)_3_ nanoparticles was examined using WST-1, LDH, and hemolysis assays. For the WST-1 assay, the HeLa cells were seeded to 1.0 × 10^4^ cells/well in a 96-well microplate and incubated for 24 h. PEI 25kD and LL, LD, DL, and DD were prepared at various concentrations (0.025 to 0.2 μg/μL). PEI 25kD, a cationic polymer, was introduced as a positive control. After 24 h of incubation, 10 µL WST-1 was added to each well and incubated for 2 h. WST-1 formazan was measured using a VERASmax microplate reader (Molecular Devices, LLC, Sunnyvale, CA, USA) at an absorbance of 450 nm. For the LDH assay, 20% Tween-20 treated cells were examined as maximum LDH activated control to calculate the LDH activity (%) of samples. After 24 h incubation, 10 μL of the supernatant of each sample was collected and added to a working reagent containing 0.2 mM NADH and 2.5 mM sodium pyruvate in a 96-well microplate and incubated for 45 min at 37 °C. Finally, the LDH activity was quantified by measuring the absorbance at 490 nm using a microplate reader.

### 2.7. Quantification of Hemolysis

The hemolysis of LL, LD, DL, and DD nanoparticles was investigated using human blood cells. PEI 25kD was introduced as a positive control. Briefly, fresh human blood was obtained from healthy volunteers. Coagulation of human blood was prevented using ethylenediaminetetraacetic acid (EDTA). The human blood was centrifuged at 5000 rpm for 5 min to separate the red blood cells (RBCs) from the serum. The isolated RBCs were washed twice using PBS and diluted to 2.0 × 10^8^ cells/mL in PBS. To evaluate the hemolytic activity, Triton X-100 0.2% treated samples were used as the maximum hemolytic activity control. A 0.25 mL volume of the prepared RBCs was mixed with 0.25 mL of each sample (final concentration of 0.1 μg/μL and 0.2 μg/μL, respectively). The mixed samples were incubated for 2 h at 37 °C. After incubation, the samples were centrifuged at 5000 rpm for 5 min, and 30 μL of the supernatant of each sample was collected. The supernatant was diluted 10-fold using PBS to a final volume of 100 μL. The prepared supernatant solution was added to a 96-well microplate and measured at 450 nm using a VERSAmax microplate reader (Molecular Devices, San Jose, CA, USA).

### 2.8. Cellular Uptake Assay

The cellular uptake ability of the peptide nanoparticle was analyzed using confocal microscopy (Zeiss International, Oberkochen, Germany) and fluorescence-activated cell sorting (FACS) (BD Biosciences, San Jose, CA). For confocal microscopy assays, 1.0 × 10^4^ cells/well were seeded in an 8-well confocal plate and incubated for 24 h. Nile Red-tagged LL, DL, DL, and DD peptide nanoparticles were treated to a final concentration of 0.2 mg/mL, and the cells were incubated for 6 h. After incubation, the cell nuclei were stained with 20 μM Hoechst 33324 for 15 min. The prepared cells were observed using confocal microscopy. In the FACS assay, 1.0 × 10^6^ cells/well were seeded in 6-well microplate and incubated for 24 h. After incubation, the Nile Red-tagged peptide nanoparticles were treated to a final concentration of 0.2 mg/mL, and cells were incubated for 16 h. After incubation, the cells were washed twice using DPBS and harvested using trypsin-EDTA. The cells were collected by centrifugation (1500 rpm, 3 min) and resuspended in 500 μL of DPBS. The prepared samples were transferred to a FACS tube and analyzed using FACS.

### 2.9. Preparation of Doxorubicin-Loaded RH-(GFLG)_3_ Nanoparticles

Doxorubicin (Dox) was encapsulated in the RH-(GFLG)_3_ peptide nanoparticles to evaluate their potential as drug delivery carriers. Doxorubicin hydrochloride (Dox-HCl) was mixed in water with excess trimethylamine to the molar ratio Dox:trimethylamine = 1:3 to desalt and was then incubated for 16 h. The desalted Dox was extracted five times using chloroform and examined to prepare the Dox-loaded RH-(GFLG)_3_ nanoparticles (Dox-LL, LD, DL, and DD). To optimize the encapsulation conditions, various molar ratios (between 0.1 and 2) of Dox to peptide were prepared. The Dox in chloroform was added in a glass vial, dried using nitrogen gas, and added to 1 μg/μL of peptide solution. The samples were vortexed, sonicated for 15 min, and incubated at 4 °C overnight. To remove the non-encapsulated Dox, the prepared Dox-loaded peptide nanoparticles were dialyzed using dialysis membrane (MWCO, 1000 Da, Spectrra/Por) for 1 day at room temperature (25 °C). The prepared Dox-loaded RH-(GFLG)_3_ nanoparticles (LL-Dox, LD-Dox, DL-Dox, and DD-Dox) were stored at 4 °C, and their size diameter and ξ-potential were measured using DLS and zeta instruments of Nano ZS90 (Malvern, Worcestershire, UK) to confirm the formulation of the nanostructure.

### 2.10. Quantitation of Doxorubicin in Nanoparticles

The doxorubicin in the peptide nanoparticles was quantified using a VERASmax microplate reader (Molecular Devices, San Jose, CA, USA). To establish the calibration curve, desalted Dox was prepared to various concentrations (0.01 to 0.5 μg/μL) in a solvent (methanol/water = 9:1, *v*/*v*) and measured at an absorbance of 480 nm. For the quantitation of Dox in the peptide nanoparticles, the Dox-loaded nanoparticles were dissolved in the same solvent (methanol/water = 9:1, *v*/*v*) and measured using a microplate reader. The drug loading efficiency and encapsulation efficiency of Dox in the peptide nanoparticles were calculated using the formulae below:

(1) $$\mathrm{Loading}\;\mathrm{efficiency}\;\left(\%\right)=\frac{\mathrm{mole}\;\mathrm{of}\;\mathrm{the}\;\mathrm{Dox}\;\mathrm{in}\;\mathrm{nanoparticles}}{\mathrm{mole}\;\mathrm{of}\;\mathrm{the}\;\mathrm{nanoparticles}}\times 100$$

(2) $$\mathrm{Encapsulation}\;\mathrm{efficiency}\;\left(\%\right)=\frac{\mathrm{mole}\;\mathrm{of}\;\mathrm{the}\;\mathrm{Dox}\;\mathrm{in}\;\mathrm{nanoparticles}}{\mathrm{mole}\;\mathrm{of}\;\mathrm{initial}\;\mathrm{Dox}}\times 100$$

### 2.11. Enzyme-Responsiveness Test

The enzyme-responsive ability of RH-(GFLG)_3_ was analyzed using matrix assisted laser mass spectroscopy (MALDI-TOF, Voyager TOF Mass Spectrometer, Applied Biosystems Inc., Carlsbad, CA, USA). The LL, LD, DL, and DD peptides were treated with 0.02 μM of the enzyme cathepsin B in an activation buffer at 37 °C for 6 h. To compare enzyme-responsive ability, control groups without enzyme were treated identically in an activation buffer at 37 °C for 6 h. The prepared peptides were measured using MALDI-TOF (Voyager TOF Mass Spectrometer) in a α-cyano-4-hydroxycinnamic acid (CHCA) matrix.

### 2.12. In Vitro Drug Release

In vitro drug release of the Dox-loaded peptide nanoparticles was examined using the dialysis method. Briefly, 300 μL of prepared Dox-loaded peptide nanoparticles (LL-Dox and DD-Dox) were added to a dialysis membrane (MWCO, 1000 Da). To determine the enzyme-responsive ability, LL-Dox and DD-Dox were treated using the enzyme cathepsin B to a final concentration of 0.02 μM in the activation buffer. The prepared dialysis bag was added to 2.7 mL PBS solution and incubated in a shaking incubator at 37 °C. The outer solution containing the released drug was collected at various incubation times of 0, 1, 3, 6, 24, 48 and 72 h and its absorbance was measured using micro reader at 480 nm.

### 2.13. Cellular Uptake of Dox-Loaded RH-(GFLG)_3_ Nanoparticles

The cellular uptake of Dox-loaded RH-(GLFG)_3_ nanoparticles in HeLa cells was analyzed using confocal microscopy. HeLa cells were seeded to 1.0 × 10^4^ cells/well in an 8-well confocal plate and incubated for 24 h. After incubation, the cells were treated using Free Dox, DD-Dox, and LL-Dox at a final concentration of 2 μM and incubated for 6 h. The nuclei of the cells were stained with 20 μM Hoechst 33324 for 15 min. The prepared cells were observed using confocal microscopy. To understand cellular uptake in tissue, we examined 3D spheroids of HeLa cells. For the formulation of the 3D spheroids, 1.0 × 10^4^ cells were seeded in a round shaped 96-well plate coated with 1% agarose and incubated for 3 days. After incubation, the cells formed a globular shaped 3D tissue. The prepared 3D spheroids were treated using Free Dox, DD-Dox, and LL-Dox at a final concentration of 1 μM. The 3D spheroid medium was changed every 2 days for the tissue growth. After 7 days, the 3D spheroids were observed using confocal microscopy.

### 2.14. Anticancer Effect of Doxorubicin-Loaded RH-(GFLG)_3_ Peptide Nanoparticles

The anticancer activity of Dox-loaded peptide nanoparticles in HeLa and SW480 cells was determined with an MTT assay. Cells were seeded to 1.0 × 10^4^ cells/well in a 96-well microplate and incubated for 24 h. After incubation, the cells were treated using Free Dox and Dox-loaded RH-(GLFG)_3_ nanoparticles to a final concentration of 0.25–2 μM and incubated for 24–72 h. After incubation, the prepared samples were analyzed following the method described above. To understand the anticancer effect in tissue, 3D spheroids of HeLa cells were prepared as described above. The prepared 3D spheroids were treated with 1 μM Free Dox, DD-Dox, and LL-Dox and incubated for 1–7 days. The morphology of the 3D spheroids was observed using an objective microscope every day. After 7 days of incubation, the viability of the spheroids was analyzed using an MTT assay. The spheroids were added to the MTT solution (2 mg/mL in DPBS) and incubated for 4 h. The supernatant was removed, and 150 μL of DMSO was added to each sample. The prepared samples were measured using a micro reader (Molecular Devices, San Jose, CA, USA) at an absorbance of 570 nm.

### 2.15. In vivo Anticancer Capability in a Zebrafish Model

To examine the anticancer activity of the Dox-loaded RH-(GLFG)_3_ nanoparticles in vivo, we generated zebrafish cancer models using the SW480 cell line. Briefly, zebrafish eggs were incubated in a fish incubator for 2 days and dechorionated. The SW480 cells were stained using cell fluorescence tracker (cell tracker green CMFDA) for 30 min in a cell culture incubator. The labeled cells were then harvested using trypsin-EDTA and washed twice with PBS containing 10% FBS. The labeled cells were then injected into zebrafish larvae by micro-injection. The larvae were split in 96-well microplates containing TAP-water. After 2 days of incubation, Free Dox and Dox-loaded RH-(GLFG)_3_ nanoparticles (LL-Dox and DD-Dox) were injected into zebrafish larvae by micro-injection. After 2 days of incubation, the larvae were anesthetized using 0.04% tricaine and observed under a fluorescence microscope (Leica DMi8, Wetzlar, Germany).

## 3. Results and Discussion

### 3.1. Preparation and Characterization of Peptide Nanoparticles

In a primary study, we prepared three types of sequences by increasing the peptide core length (RH-(GFLG)_1_, RH-(GFLG)_2_, and RH-(GFLG)_3_) to optimize the formation of peptide nanoparticles (Table S1). These peptides were designed to include a hydrophilic head (Arg and His) and a repeating hydrophobic tail (Gly-Lue-Phe-Gly) of enzyme-responsive oligopeptides. Moreover, we introduced the enantiomer peptides ^L^RH-^D^(GFLG)_3_ (LD), ^D^RH-^L^(GFLG)_3_ (DL), and ^D^RH-^D^(GFLG)_3_ (DD) as negative control to analyze the enzyme sensitivity of the RH-(GFLG)_3_ peptide nanoparticles (Table S2). The peptides were synthesized by solid-phase peptide synthesis, purified using high performance liquid chromatography (HPLC), and characterized and confirmed by matrix assisted laser mass spectroscopy (MALDI-MS) (Figure S1). The HPLC data presented an elution peak at 7.5 min of retention time. The MALDI-MS spectrum showed a sharp peak at 1433.08 *m*/*z*, which was similar to the expected mass of 1433.76. These results prove that the peptides were successfully synthesized.

As previously mentioned, arginine is a representative cell-penetrating peptide that can enhance cellular uptake [6]. Histidine incorporating an imidazole group that possesses a proton buffering capacity can induce endo/lysosomal escape after endocytosis [40]. The repeating GLFG peptides provide a hydrophobic core containing phenylalanine. The phenyl group of phenylalanine induces the formation of stable nanoparticles and has been applied to various peptide self-assemblies [41]. The GLFG peptide can cleave cathepsin B by endo-peptidase, indicating that the GLFG responsive system can induce both endosome escape and release of cargo molecules [42]. Thus, recent studies have reported effective delivery systems using GLFG peptides [43,44]. Specifically, it has been shown that the cathepsin B enzyme is related to the development of cancer and that a cathepsin B-responsive delivery system has potential for chemotherapy [45].

The diameter of the RH-(GFLG)_1_ and RH-(GFLG)_2_ peptide nanoparticles was too small to be measured, indicating these peptide nanostructures are not formed easily because of their low hydrophobicity. However, RH-(GFLG)_3_ formed nano-sized particles of 118.4 ± 7.7 nm with a positive ξ-potential of 30.1 mV. These results suggest that RH-(GFLG)_3_ could form self-assembled nanoparticles with a high positive surface charge. Therefore, further studies were performed to evaluate the potential of RH-(GFLG)_3_ as a drug delivery carrier. In addition, peptides ^L^RH-^D^(GFLG)_3_ (LD), ^D^RH-^L^(GFLG)_3_ (DL), and ^D^RH-^D^(GFLG)_3_ (DD) were used as non-enzyme- sensitivity controls to determine the enzyme-sensitive effect of ^L^RH-^L^(GFLG)_3_ (LL).

**Figure 2:**
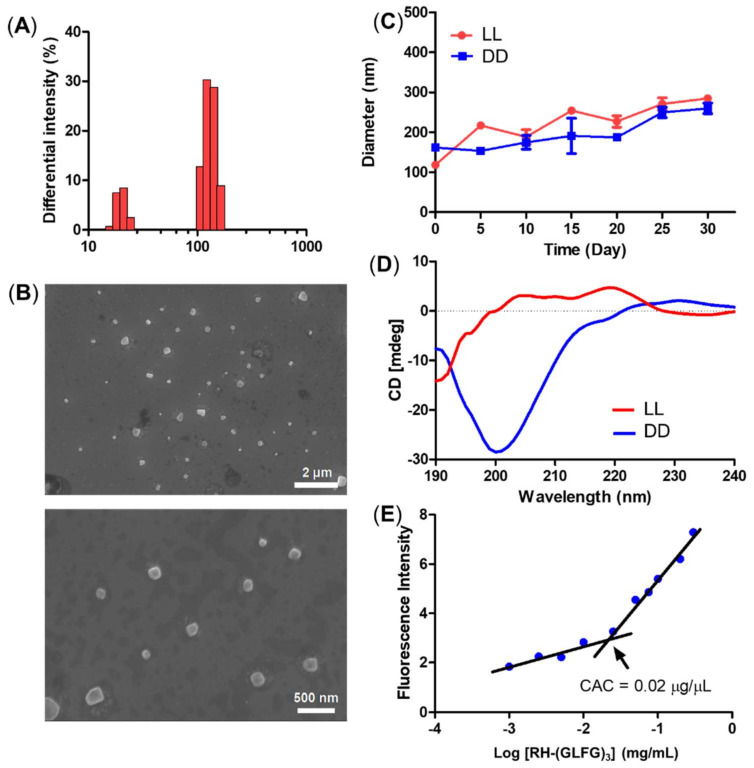
Physical characterization of the RH-(GFLG)_3_ nanoparticles. (**A**) Size distribution of LL nanoparticles. (**B**) Field emission-scanning electron microscopy image. (**C**) Size change of LL and DD nanoparticles for a storage time of 30 days. The size distribution was measured every 5 days. Values are reported as mean ± SEM (*n* = 3). (**D**) Circular dichroism (CD) spectrum of LL and DD nanoparticles. (**E**) Critical aggregation concentration (CAC) of LL nanoparticles.

The size distribution of the LL nanoparticle presented a small and relatively uniform histogram with sizes around 10 and 100 nm (Figure 2A). The morphology of the LL nanoparticles was observed using FE-SEM (Figure 2B). The FE-SEM images showed a globular and uniform nanostructure smaller than 100 nm. To determine the stability of the peptide nanoparticles, we monitored the diameter of LL and DD samples for 30 days at 4 °C (Figure 2C). Both LL and DD maintained their nanoscale diameter of less than 200 nm during the 30 days. These results suggest that the RH-(GFLG)_3_ nanoparticles have good stability owing to the hydrophobic core of phenylalanine. The self-assembled RH-(GFLG)_3_ nanoparticles were analyzed using CD spectroscopy (Figure 2D). The LL nanoparticles showed high turn organization (turn 91.5% and random 8.5%), generated by repeated GLFG peptide sequences. A random structure of 8.5% indicates peptide nanoparticles that do not form a self-assembled structure. We speculate that these are colloidal particles with a size in the 10 nm range observed in the DLS and FE-SEM analysis. However, the DD nanoparticles showed a complex structure with a high random ratio (beta 29.2%, turn 15%, and random 55.8%) due to the ^D^RH-^D^(GFLG)_3_ (DD) enantiomer peptide. D-form peptides have different light polarization compared with L-form peptides [46]; therefore, the secondary structure of DD peptides had a different CD spectrum than the LL peptides despite having the same peptide sequence. As a result, both the LL and DD peptide nanoparticles possess a secondary structure based on self-assembly. In addition, we analyzed the CAC of RH-(GFLG)_3_ peptide nanoparticles using Nile Red (Figure 2E). The CAC value is one of the characteristics of self-assembled structures. This method is based on the spectral shift of a fluorescence probe in a hydrophobic environment. At RH-(GFLG)_3_ peptide nanoparticle concentrations lower than 0.02 mg/mL, Nile Red showed reduced fluorescence intensity. Therefore, the CAC value of RH-(GFLG)_3_ peptide nanoparticles was found to be around 0.02 mg/mL. Overall, the RH-(GFLG)_3_ nanoparticles have potential as drug delivery carriers due to their stable self-assembled formulation.

### 3.2. Cytotoxicity of RH-(GFLG)_3_ Peptide Nanoparticles

**Figure 3:**
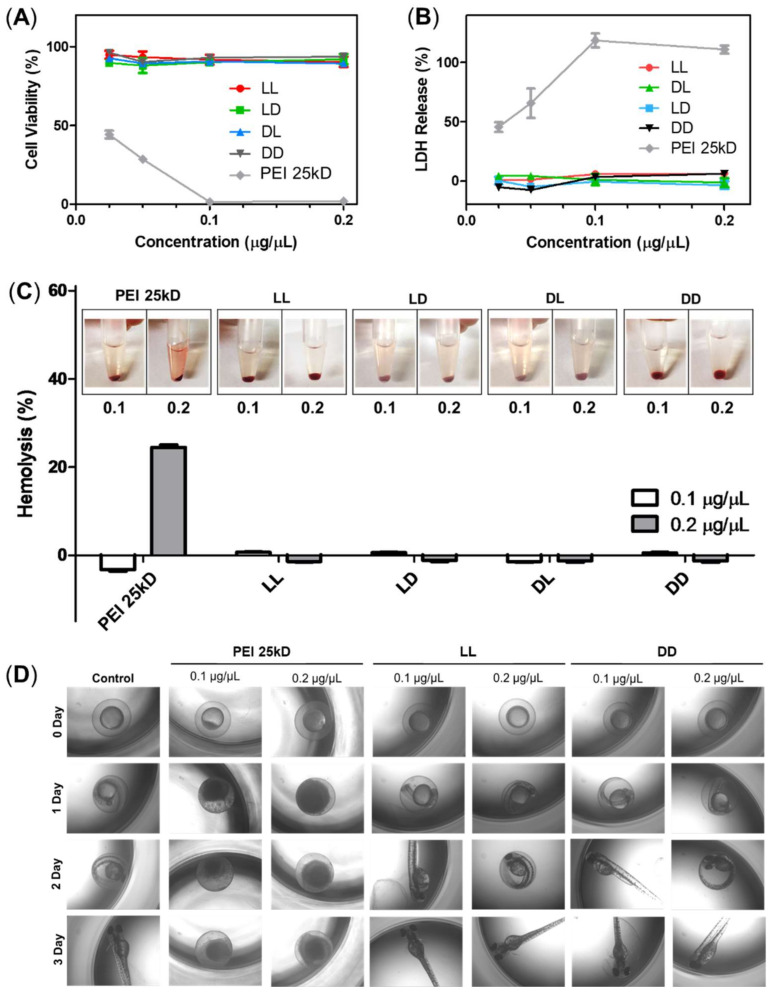
Cytotoxicity of RH-(GFLG)_3_ nanoparticles. Cell viability using (**A**) MTT assay and (**B**) LDH assay. Concentrations of 0.2, 0.1, 0.5, and 0.25 µg/µL were tested for an incubation time of 24 h in HeLa cells. Values are reported as mean ± SEM (*n* = 3). (**C**) Hemolysis assay testing concentrations of 0.1 and 0.2 µg/µL using human blood cells. PEI 25kD was used as positive control. Values are reported as mean ± SEM (*n* = 3). (**D**) Zebrafish embryo test performed with nanoparticles at concentrations of 0.1 and 0.2 µg/µL. The incubation time was 3 days.

The cytotoxicity of RH-(GFLG)_3_ was analyzed using MTT, LDH, and hemolysis assays. We also examined *b*-PEI, a representative cationic carrier, as positive control for comparison with RH-(GFLG)_3_ peptide nanoparticles. In the MTT assay, the LL, LD, DL, and DD RH-(GFLG)_3_ nanoparticles showed negligible cytotoxicity at the highest concentration of 0.2 μg/μL, whereas *b*-PEI presented high toxicity below 0.025 μg/μL (Figure 3A). The LDH assay was used to demonstrate membrane damage caused by the RH-(GFLG)_3_ nanoparticles (Figure 3B). Similarly to the MTT assay, the LL, LD, DL, and DD RH-(GFLG)_3_ nanoparticles showed negligible LDH release, whereas *b*-PEI exhibited high LDH release of up to 50% at concentrations of up to 0.05 μg/μL. Since hemolysis can improve red blood cell (RBC) membrane damage through interaction with certain molecules (Figure 3C), it is an important criterion for biomedical applications, while its negligible occurrence is advantageous for a safe delivery system. The RH-(GFLG)_3_ nanoparticles showed negligible hemolysis when applied to human RBCs, whereas *b*-PEI presented extreme hemolytic activity of approximately 20% at 0.2 μg/μL. These results indicated that the RH-(GFLG)_3_ nanoparticles have no hemolytic activity compared to the untreated control group. We applied an embryo toxicity test with the zebrafish to evaluate cytotoxicity in vivo (Figure 3D). Zebrafish embryos are sensitive to toxic materials during their development, and the physical effects of toxicity on their growth and evolution can be analyzed experimentally [47,48]. In the *b*-PEI samples, the eggs did not develop normally into larvae due to b-PEI‘s toxic effect. However, in the RH-(GFLG)_3_ control group, the zebrafish eggs showed healthy development. These results indicate that the RH-(GFLG)_3_ nanoparticles have low cytotoxicity in in vivo systems.

The cytotoxicity analysis proved that the RH-(GFLG)_3_ nanoparticles have excellent biocompatibility and can minimize cellular cytotoxicity in the delivery system.

### 3.3. Preparation of Doxorubicin-Loaded RH-(GFLG)_3_ Nanoparticles

Doxorubicin (Dox), which is a common anticancer drug, was encapsulated into the RH-(GFLG)_3_ nanoparticles to evaluate their drug delivery ability. Dox has been used in the treatment of various tumors, such as breast cancer, bladder cancer, and lymphoma cancer. In addition, recent studies reported that Dox-treated cancer cells showed upregulation of cathepsin B. The combination of RH-(GFLG)_3_ nanoparticles and Dox can induce effective drug release via the increased concentrations of cathepsin B. To identify the ideal encapsulation conditions, Dox was prepared at various molar ratios with respect to the peptide monomer (peptide:Dox = 1:0.1–3, molar ratio) (Table S3). The Dox-loaded RH-(GFLG)_3_ nanoparticles presented a larger diameter than the empty RH-(GFLG)_3_ nanoparticles since Dox was encapsulated inside the hydrophobic core of the nanoparticles. The Dox-loaded RH-(GFLG)_3_ with 0.1 and 0.5 molar ratios had an unstable formulation with a relatively large diameter cause by aggregation, whereas Dox-loaded RH-(GFLG)_3_ nanoparticles with 1–3 molar ratios had a diameter of 170–180 nm. The ξ-potential of the Dox-loaded RH-(GFLG)_3_ showed a high cationic charge of approximately 30 mV, similar to that of the empty RH-(GFLG)_3_ nanoparticles. The high cationic charge of the Dox-loaded RH-(GFLG)_3_ indicated a maintained self-assembled structure. The Dox-loaded RH-(GFLG)_3_ of 0.1–2 molar ratios showed increased loading and encapsulation efficiencies with increased doxorubicin amounts. However, the 1:3 peptide to Dox ratio showed loading efficiencies similar to those of the 1:2 ratio, indicating that the 1:2 ratio is the maximum condition to encapsulate doxorubicin. Considering the loading efficiency and diameter of Dox-loaded RH-(GFLG)_3_ nanoparticles, we chose the 1:2 ratio as the ideal encapsulating condition, which was subsequently used in the evaluation of the anticancer effect.

### 3.4. Cellular Uptake Assay

**Figure 4:**
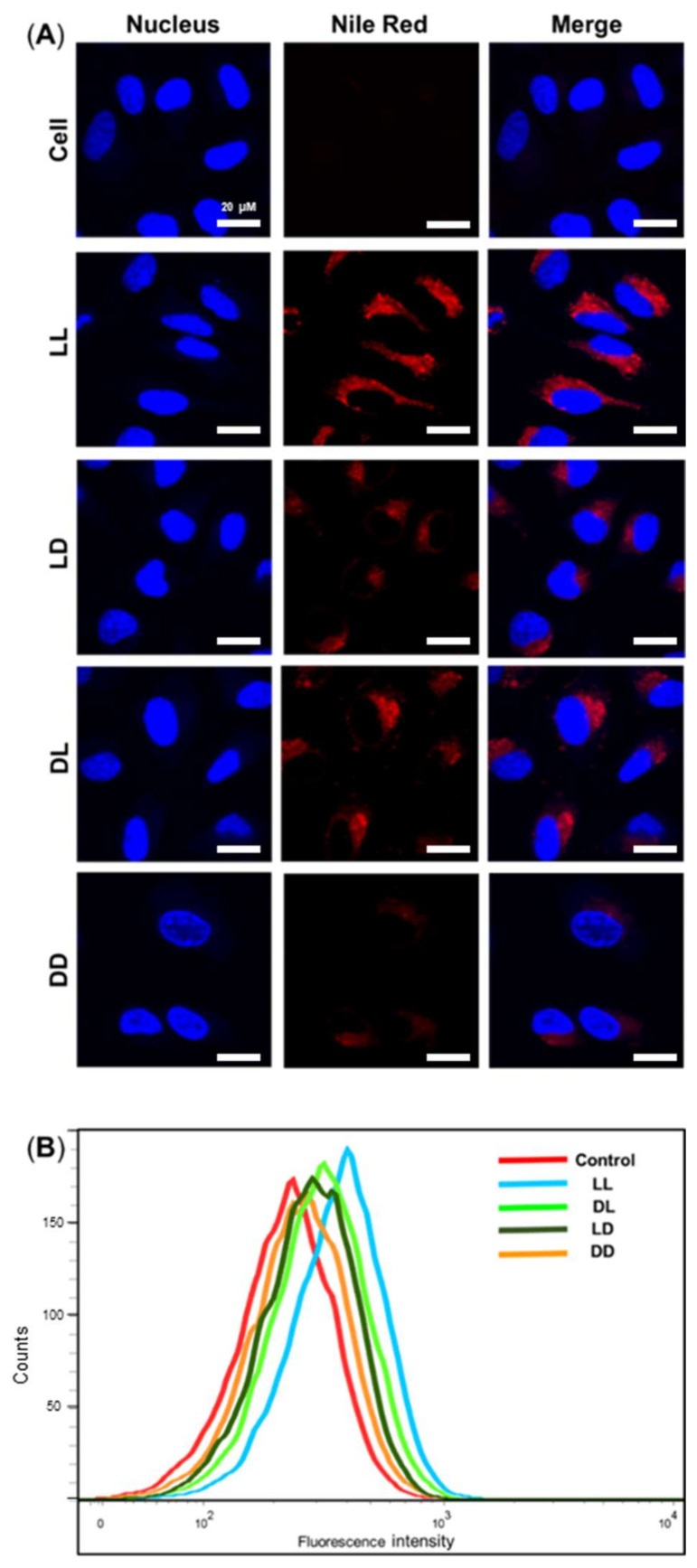
Cellular uptake assay of the Nile Red-tagged RH-(GFLG)_3_ nanoparticles. (**A**) Fluorescence images obtained via confocal microscopy of cell, LL, LD, DL, and DD following incubation for 6 h. Scale bar = 20 µm (blue = nucleus, red = RH-(GFLG)_3_ nanoparticles). (**B**) Fluorescence-activated cell sorting (FACS) analysis of cells incubated with control (empty peptide nanoparticles), LL, LD, DL, and DD during an incubation time of 16 h.

The cellular uptake of Nile Red-tagged RH-(GFLG)_3_ nanoparticles in HeLa cells was analyzed by confocal microscopy and FACS (Figure 4). After 6 h of incubation, LL, LD, DL, and DD showed different uptake ability. The LL nanoparticles showed higher uptake compared to LD, DL, and DD. The LD and DL presented similar uptake abilities, and DD had the lowest uptake ability (LL > LD = DL > DD). These results are related to the peptide sequence isomer. The LD nanoparticles did not exhibit an enzyme-responsive ability owing to their GFLG isomer core; the DL nanoparticle presented less uptake ability due to D-arginine. Therefore, the DD nanoparticles had the lowest cellular uptake ability. The difference in uptake ability among the RH-(GFLG)_3_ nanoparticles is expected to affect drug delivery efficiency. In addition, RH-(GFLG)_3_ proved to have good cellular uptake ability because of its enzyme-sensitive character, which is a useful feature in a delivery system.

### 3.5. Enzyme Sensitivity Test

**Figure 5:**
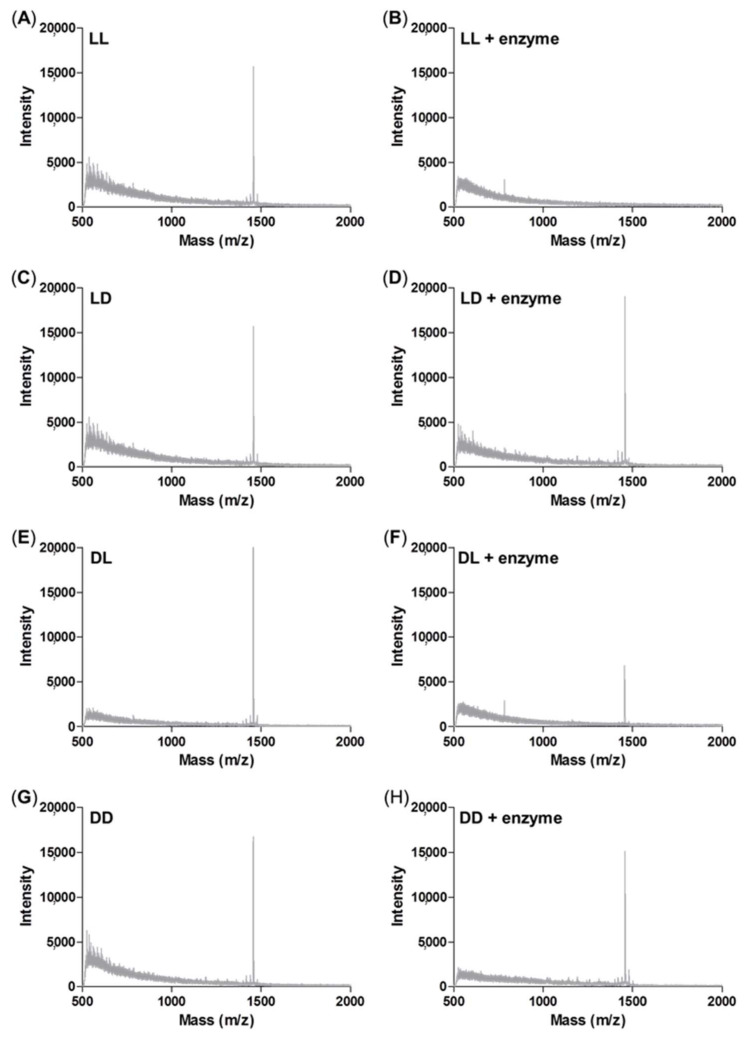
Enzyme sensitivity assay using MALDI-TOF mass spectrometry analysis. (**A**) LL peptide control; (**B**) LL peptide by cathepsin B treatment; (**C**) LD peptide control; (**D**) LD peptide by cathepsin B treatment; (**E**) DL peptide control; (**F**) DL peptide by cathepsin B treatment; (**G**) DD peptide control; (**H**) DD peptide by cathepsin B treatment.

We designed an enzyme-responsive peptide nanoparticle modified with a GLFG core. As mentioned above, isomers containing LD, DL, and DD peptides were used as control groups. The enzyme sensitivity was analyzed using an enzyme treatment and MALDI-TOF MS (Figure 5). Each peptide was treated with cathepsin B, and the degree of degradation was analyzed by mass spectrometry.

The original mass spectra of the non-enzyme-treated groups of LL, LD, DL, and DD showed peaks at around 1433 *m*/*z* as shown in Figure 5A,C,E,G. whereas the mass spectra of the enzyme-treated LL peptide showed no peak at 1433 *m*/*z* (Figure 5B), which indicates the degradation of the peptide by enzyme (Figure 5B). The enzyme-treated DL peptide presented reduced intensity at 1433 *m*/*z* (Figure 5F). This result indicated that the repetitive GLFG unit of the DL peptide was degraded. In contrast, the mass spectra of the enzyme-treated LD and DD were unchanged because the isomer of the GLFG core cannot be degraded by the enzyme cathepsin B (Figure 5D,H). Through the enzyme sensitivity test using mass, the GLFG core was shown to generate an enzyme-responsive ability. In addition, these results indicated that RH-(GFLG)_3_ nanoparticles have the potential for effective drug release by the enzyme cathepsin B.

### 3.6. In Vitro Drug Release

**Figure 6:**
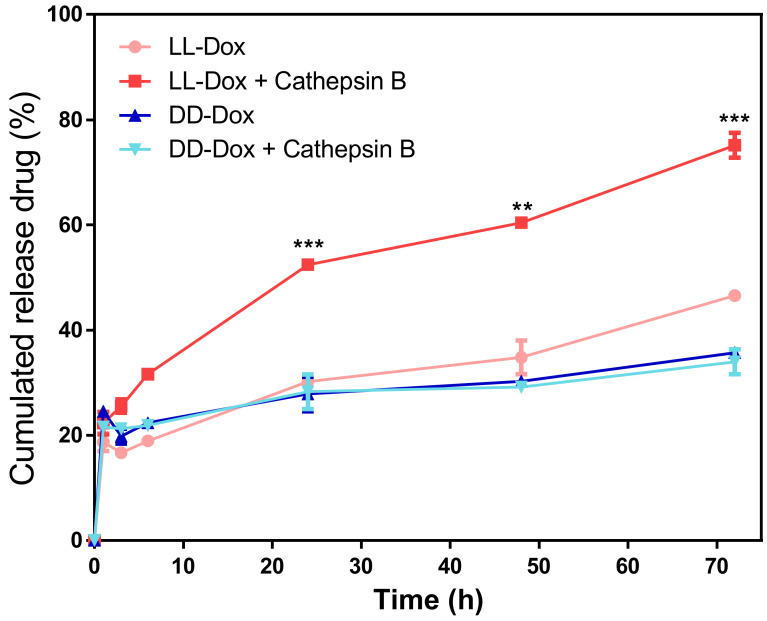
In vitro drug release test of LL-Dox and DD-Dox by enzyme treatment. Tested incubation times were 0, 1, 3, 6, 24, 48, and 72 h. Values are reported as mean ± SEM (*n* = 3). Statistical analysis was performed using Student’s *t*-test, ** *p* < 0.01, *** *p* < 0.001 versus LL-DOX.

The drug release capability of RH-(GFLG)_3_ was determined using a dialysis method and microreader at 480 nm. To confirm the enzyme-sensitive activity of the RH-(GFLG)_3_ nanoparticles, we prepared enzyme-treated and non-treated LL and DD-Dox (Figure 6). DD-Dox showed approximately 20% drug release in both the enzyme-treated and non-treated samples. This result suggests that the isomer of the GLFG core does not respond to the enzyme cathepsin B, resulting in low drug release. However, the LL-Dox was responsible for the difference between the enzyme-treated and non-treated samples. The L-Dox-treated with the enzyme showed higher drug release (approximately 80%) compared to the non-treated samples, indicating that the LL peptide is enzyme-sensitive and the degradation of the nanoparticle by the enzymes promotes drug release. The enzyme sensitivity of the RH-(GFLG)_3_ nanoparticles can thus affect drug delivery efficiency after intracellular uptake. 

### 3.7. Cellular Uptake of Dox-Loaded RH-(GFLG)_3_ Nanoparticles in 3D Spheroids

**Figure 7:**
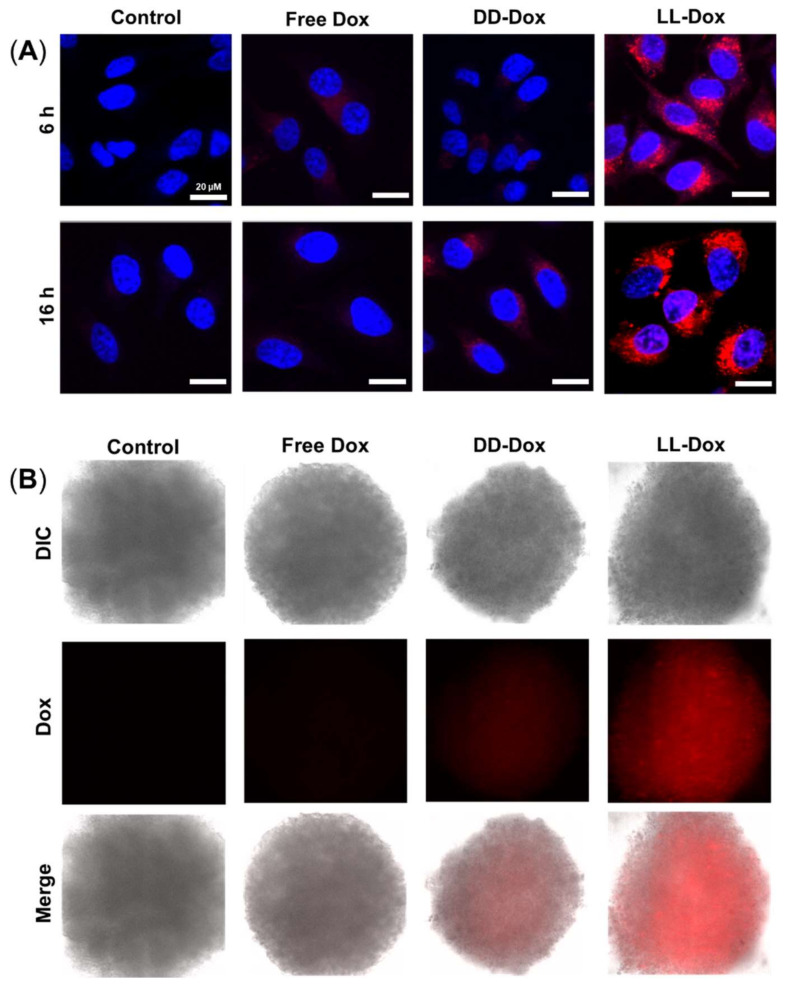
Cellular uptake assay of Dox-loaded RH-(GFLG)_3_ nanoparticles. (**A**) Fluorescence images of HeLa cells obtained using confocal microscopy (blue = nucleus, red = doxorubicin). Scale bar = 20 µm. (**B**) Fluorescence images of the 3D spheroid model in HeLa cells using fluorescence microscopy (red = doxorubicin).

The cellular uptake of Dox-loaded RH-(GFLG)_3_ nanoparticles in HeLa cells and 3D spheroids was analyzed using confocal microscopy (Figure 7). Doxorubicin with red fluorescence was used in the cellular uptake assay. The cells were treated with 1 μM Free Dox, DD-Dox, and LL-Dox and were observed after an incubation time of 16 h. Free Dox and DD-Dox had a low cellular uptake ability. However, LL-Dox presented a higher cellular uptake ability than Free Dox and DD-Dox. Similarly to the assay with HeLa cells, LL-Dox showed an excellent cellular uptake ability in the 3D spheroid model. Through the cellular uptake of Dox, the high cellular uptake ability of RH-(GFLG)_3_ is expected to influence the anticancer effect.

### 3.8. Anticancer Effect of Doxorubicin-Loaded RH-(GFLG)_3_

**Figure 8:**
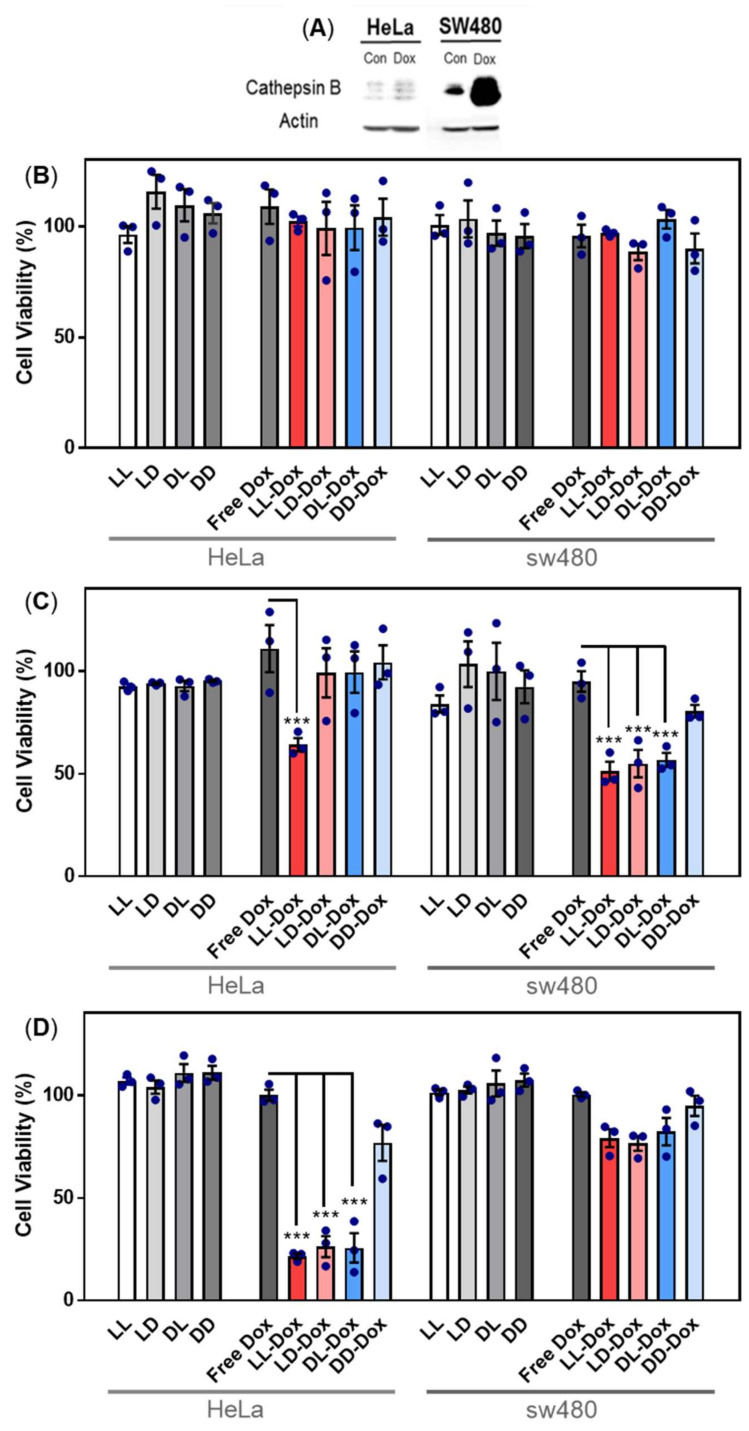
Anticancer activity of Dox-loaded RH-(GFLG)_3_ in HeLa and SW480 cells. (**A**) Protein expression level of cathepsin B in HeLa and SW480 cells by Dox treatment. Viability of (**B**) 24 h incubation, (**C**) 48 h incubation, and (**D**) 72 h incubation. Concentration of Dox is 500 nM. Values are presented as mean ± SEM (*n* = 3). Blue circles of each sample are individual results. Statistical analysis performed by one-way ANOVA, *** *p* < 0.001 versus free-Dox.

To determine the anticancer effect of Dox-loaded RH-(GFLG)_3_, we performed cell viability and 3D spheroid assays. Dox as a representative anticancer drug was applied to evaluate the drug delivery efficiency of RH-(GFLG)_3_. In a primary test, we treated HeLa cell with various concentrations of Dox, (Free Dox, LL-Dox, and DD-Dox) ranging from 0.25 to 2 μM (Figure S2). The concentration of peptide control groups was defined at 2 μM Dox, which exhibited negligible cytotoxicity, implying that the peptide did not influence the anticancer effect of DD-Dox and LL-Dox. DD-Dox showed a slow anticancer effect for over 3 days at concentrations of 0.5 to 2 μM. In contrast, LL-Dox showed anticancer effects from day 1 and continued to show excellent effects even at low concentrations of 0.25 μM until day 3. These results demonstrate that LL-Dox achieved a high anticancer effect due to controlled release of the drug following the enzyme-responsive modification. DD-Dox also showed a gradual anticancer effect over the peptide hydrolysis for 3 days. The findings indicated that the GLFG peptide core induced a fast and effective drug release compared to the control groups. To identify the drug release ability of RH-(GFLG)_3_ nanoparticles depending on the cathepsin B level, we conducted a comparative analysis between HeLa and SW480 cells. SW480 cells, colon cancer cells in which cathepsin B is upregulated, were selected to analyze enzyme sensitivity [49]. First, the cathepsin B expression level in HeLa and SW480 was confirmed using Western blotting (Figure 8A). Moreover, we prepared Dox-treated cells to confirm cathepsin B expression level by Dox treatment. Cathepsin B in SW480 presented a higher expression level than in HeLa cells. Dox-treated HeLa and SW480 showed an increased expression level compared to control groups. Especially, SW480 showed a high expression level of cathepsin B by treatment with Dox, thus showing potential for fast and effective drug release. The 500 nM of Dox-loaded samples (LL-Dox, LD-Dox, DL-Dox, and DD-Dox) were incubated for 24, 48, and 72 h in HeLa and SW480 cells (Figure 8B–D). Peptide control groups (LL, LD, DL, and DD) showed negligible cytotoxicity in all conditions. No anticancer effect was observed at the 24 h timepoint, in both HeLa and SW480 cells (Figure 8B). After 48 h of incubation, LL-Dox in HeLa and SW480 cells produced a significant anticancer effect. At the 72 h timepoint, LL-Dox, LD-Dox, and DL-Dox, but not DD-Dox, induced significant anticancer effects in HeLa cells. In contrast, none of the conditions produced an anticancer effect in SW480 at this timepoint. These findings suggest that Dox was released by high cathepsin B levels in SW480. The Dox effect occurred rapidly, within 48 h, whereas recovery of cell viability presented at the 72-h incubation time.

**Figure 9:**
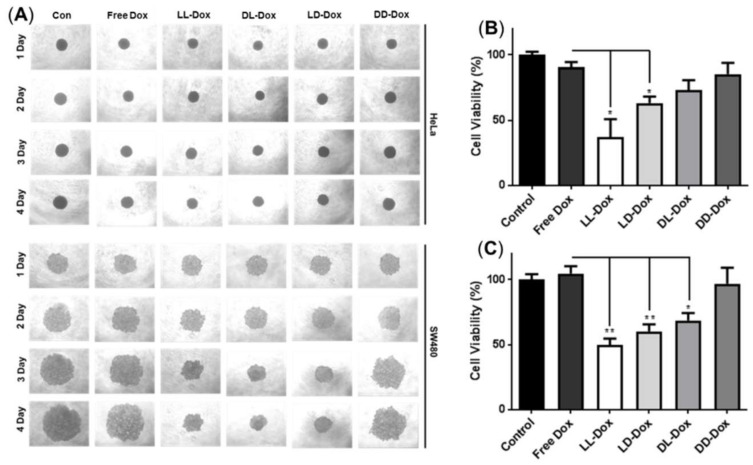
Anticancer activity of Dox-loaded RH-(GFLG)_3_ in 3D spheroids. (**A**) Images of spheroids of HeLa and SW480 cells for 4 days. (**B**) MTT assay in 3D spheroids of HeLa cells. (**C**) MTT assay in spheroids of SW480. Values are reported as mean ± SEM (*n* = 3). Statistical analysis by one-way ANOVA, * *p* < 0.05, ** *p* < 0.01 versus Free Dox.

To confirm anticancer ability in tissue conditions, we introduced a 3D spheroid model of HeLa and SW480 cells (Figure 9). The 3D spheroid model was well-formed in the HeLa and SW480 control groups (Figure 9A). Following 4 days of incubation, we evaluated daily morphological changes of the spheroids. In the 3D spheroids of HeLa cells, there were no changes in morphology or size. However, MTT assay results revealed that LL-Dox and LD-Dox showed anticancer effects after 4 days of incubation (Figure 9B). It appears that the density of the spheroids was reduced due to the anticancer effect of Dox-loaded RH-(GFLG)_3_. In the 3D spheroids of SW480 cells, LL-Dox, LD-Dox, and DL-Dox decreased in accordance with the incubation time. Cell viability data also showed that LL-Dox, LD-Dox, and DL-Dox had anticancer effects. Moreover, LL-Dox showed a higher anticancer effect than LD-Dox and DL-Dox in both HeLa and SW480. This was due to the effective drug release ability of LL-Dox. The 3D spheroid model was consistent with the cell experiment results and demonstrated the anticancer effect of RH-(GFLG)_3_ nanoparticles in tissue.

The anticancer effects, verified using cell and 3D spheroids, indicate that RH-(GFLG)_3_ peptide nanoparticles show an early and fast drug release by cathepsin B enzyme responsiveness.

### 3.9. In Vivo Anticancer Capability in a Zebrafish Model

**Figure 10:**
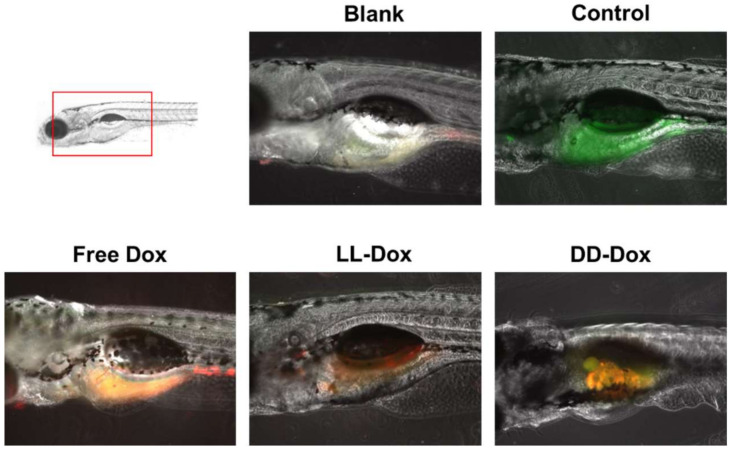
In vivo zebrafish assay used to assess the anticancer activity of Dox-loaded RH-(GFLG)_3_ nanoparticles. Images of cell tracker SW480 in zebrafish larvae (green: cell tracker, red: Dox).

To identify the anticancer capability of Dox-loaded RH-(GFLG)_3_ nanoparticles in vivo, we generated the zebrafish cancer model with SW480 cells (Figure 10). Control showed SW480 cells stained with green signal in the zebrafish yolk. Free Dox presented a merged signal of cell and Dox, illustrating that Free Dox remained in the cancer cells while exhibiting a low anticancer effect. LL-Dox effectively inhibited cancer cell growth, showing a decreased green fluorescence signal. However, DD-Dox showed a merged signal of cancer cells and puncta fluorescence, which could be from encapsulated drugs due to low targeting and reduced release from the peptide isomers. Therefore, RH-(GFLG)_3_ nanoparticles have the potential to deliver drugs by enzyme responsiveness for in vivo cancer therapy.

## 4. Conclusions

In summary, we designed enzyme-responsive nanoparticles RH-(GFLG)_3_ with a cathepsin B-sensitive core of GLFG oligopeptides. The RH-(GFLG)_3_ nanoparticles, as a novel drug delivery system, exhibited excellent biocompatibility, biodegradability, and controlled drug release by enzyme. The RH-(GFLG)_3_ peptides containing arginine, histidine, and the enzyme-sensitive core exhibited a stable nano-size formulation and enzyme-responsive property. The formulated RH-(GFLG)_3_ nanoparticles showed enhanced cellular uptake ability compared to the non-enzyme-responsive control. In addition, the RH-(GFLG)_3_ nanoparticles revealed negligible cytotoxicity in HeLa cells in the MTT, LDH, and hemolysis assays. Doxorubicin-loaded RH-(GFLG)_3_ nanoparticles showed robust anticancer ability. LL-Dox showed an improved anticancer effect compared to the control groups containing isomer, which improves enzyme-responsive property to facilitate effective drug release. Moreover, LL-Dox presented an excellent anticancer effect in a 3D spheroid tissue model and zebrafish cancer model. Based on these results, we conclude that RH-(GFLG)_3_ nanoparticles are a novel drug delivery carrier with high drug delivery efficiency and biocompatibility for biomedical applications.

## Supplementary Materials

The following are available online at https://www.mdpi.com/article/10.3390/pharmaceutics14010143/s1, Figure S1: Results of high-performance liquid chromatography and mass spectrum of matrix assisted laser mass spectroscopy of (A) ^L^RH-^L^(GLFG)_3_ (LL), (B) ^L^RH-^D^(GLFG)_3_ (LD), (C) ^D^RH-^L^(GLFG)_3_ (DL), and (D) ^D^RH-^D^(GLFG)_3_ (DD) peptide monomers; Figure S2: Anticancer activity of RH-(GFLG)_3_ in HeLa cells. Viability of (A) 24 h incubation, (B) 48 h incubation, and (C) 72 h incubation. Values are reported as mean ± SEM (*n* = 3). Statistical analysis by one-way Anova, * *p* < 0.05, ** *p* < 0.01, and *** *p* < 0.001 versus free-Dox; Table S1: Physical characterization of the RH-GLFG_3_ nanoparticles. Values are reported as mean ± SEM (*n* = 3); Table S2: Physical characterization of ^L^RH-^L^(GLFG)_3_ (LL), ^L^RH-^D^(GLFG)_3_ (LD), ^D^RH-^L^(GLFG)_3_ (DL), and ^D^RH-^D^(GLFG)_3_ (DD). Values are reported as mean ± SEM (*n* = 3); Table S3: Physical characterization of the RH-(GFLG)_3_ nanoparticles with respect to the concentration of doxorubicin. Values are reported as mean ± SEM (*n* = 3).
