# Supplementary material for: Enzyme-Responsive Amphiphilic Peptide Nanoparticles for Biocompatible and Efficient Drug Delivery
Source: Pharmaceutics. 2022 Jan 7;14(1):143. doi: 10.3390/pharmaceutics14010143 (PMC8779831; doi:10.3390/pharmaceutics14010143)
Supplement: Supplementary file 1 [file pharmaceutics-14-00143-s001.zip › pharmaceutics-1521323-supplementary.pdf]

pharmaceutics

Supplementary Materials: Enzyme-Responsive Amphiphilic Peptide Nanoparticles for Biocompatible and Efficient Drug Delivery

Su Jeong Song and Joon Sig Choi *

Table S1. Physical characterization of the RH-(GLFG)$_3$ nanoparticles. Values are reported as mean ± SEM (n=3).

Peptide	Hydrodynamic diameter (mm)	Polydispersity index (PDI)	Ξ-Potential
RH-(GFLG)$_1$	N/A	N/A	-
RH-(GFLG)$_2$	N/A	N/A	-
RH-(GFLG)$_3$	118.4 ± 7.7	0.245 ± 0.010	30.1 ± 0.4

Table S2. Physical characterization of the ^{L}RH-^{L}(GLFG)$_3$ (LL), ^{L}RH-^{D}(GLFG)$_3$ (LD), ^{D}RH-^{L}(GLFG)$_3$ (DL), and ^{D}RH-^{D}(GLFG)$_3$ (DD). Values are reported as mean ± SEM (n=3).

Peptide	Hydrodynamic diameter (mm)	Polydispersity index (PDI)	Ξ-Potential
^{L}RH-^{L}(GFLG)$_3$ (LL)	118.4 ± 7.7	0.245 ± 0.010	30.1 ± 0.4
^{L}RH-^{D}(GFLG)$_3$ (LD)	80.4 ± 1.5	0.531 ± 0.127	40.2 ± 0.8
^{D}RH-^{L}(GFLF)$_3$ (DL)	112.7 ± 7.9	0.325 ± 0.010	33.0 ± 1.5
^{D}RH-^{D}(GFLG)$_3$ (DD)	162.0 ± 7.9	0.303 ± 0.033	29.7 ± 1.3

Table S3. Physical characterization of the ^{L}RH-^{L}(GLFG)$_3$ (LL), ^{L}RH-^{D}(GLFG)$_3$ (LD), ^{D}RH-^{L}(GLFG)$_3$ (DL), and ^{D}RH-^{D}(GLFG)$_3$ (DD). nanoparticles with respect to the concentration of doxorubicin. Values are reported as mean ± SEM (n=3).

Formulation condition (molar ratio)	Hydrodynamic size (mm)	Ξ-Potential (mV)	Loading efficiency (%)	Encapsulation efficiency (%)
LL/Dox (1:0.1)	470.2 ± 102.0	31.5 ± 5.6	0.82 ± 0.02	8.22 ± 0.26
LL/Dox (1:0.5)	335.0 ± 133.4	30.7 ± 2.9	2.90 ± 0.07	5.79 ± 0.15
LL/Dox (1:1)	189.6 ± 6.6	35.9 ± 1.6	5.79 ± 0.15	5.79 ± 0.11
LL/Dox (1:2)	168.2 ± 2.3	36.5 ± 4.8	12.46 ± 0.011	6.23 ± 0.06
LL/Dox (1:3)	170.8 ± 3.6	35.4 ± 3.2	12.90 ± 0.41	4.03 ± 0.14
LD/Dox (1:2)	185.6 ± 2.4	33.7 ± 2.1	11.78 ± 0.63	5.33 ± 0.09
DL/Dox (1:2)	179.3 ± 3.9	34.7 ± 3.1	12.16 ± 0.55	5.96 ± 0.20
DD/Dox (1:2)	180.6 ± 6.3	32.7 ± 3.7	9.61 ± 0.15	4.70 ± 0.07

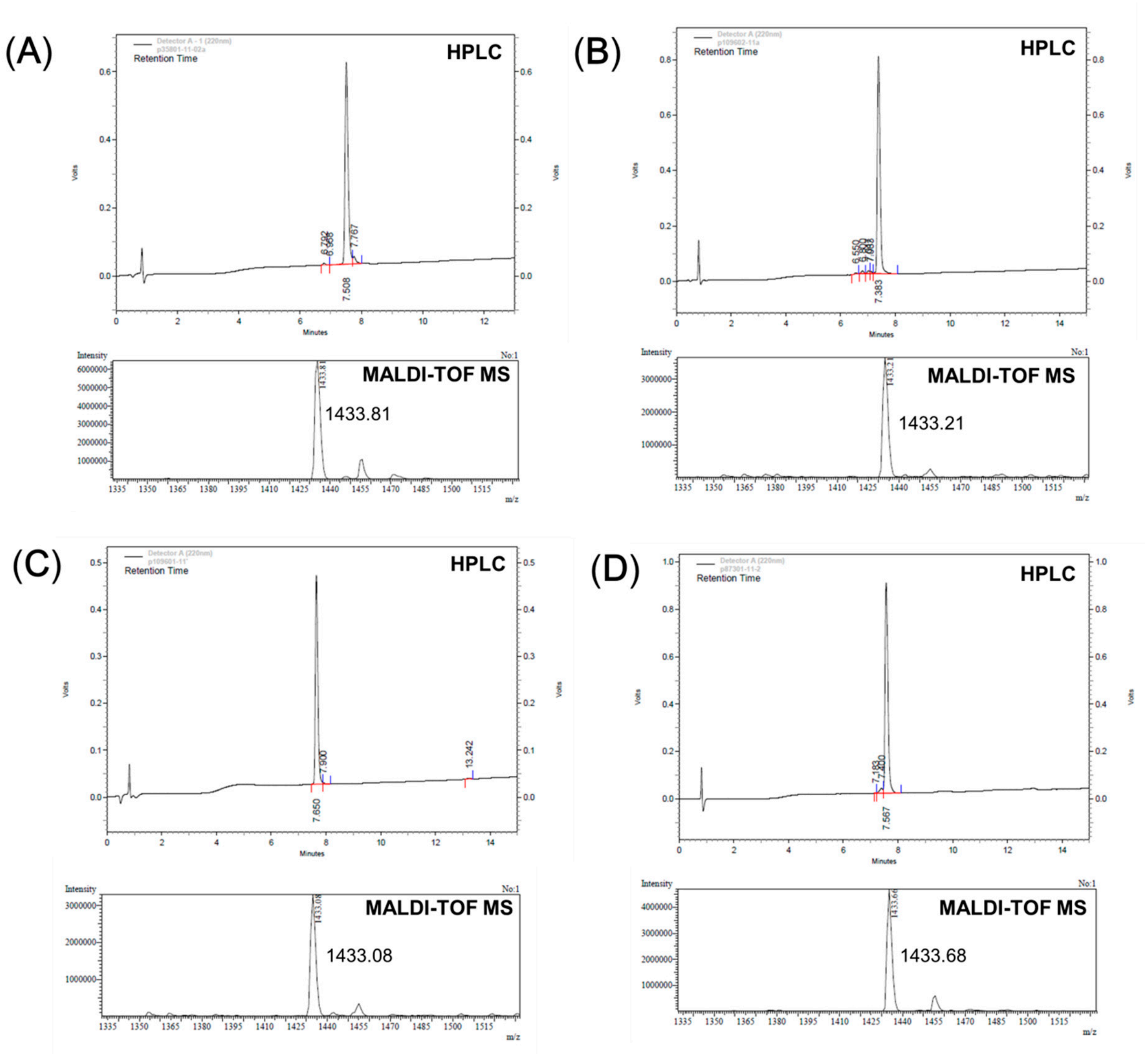

Figure S1. Results of high performance liquid chromatograph and mass spectrum of matrix assisted laser mass spectroscopy of (A) ^{L}RH-^{L}(GLFG)$_3$ (LL), (B) ^{L}RH-^{D}(GLFG)$_3$ (LD), (C) ^{D}RH-^{L}(GLFG)$_3$ (DL), and (D) ^{D}RH-^{D}(GLFG)$_3$ (DD) peptide monomers.

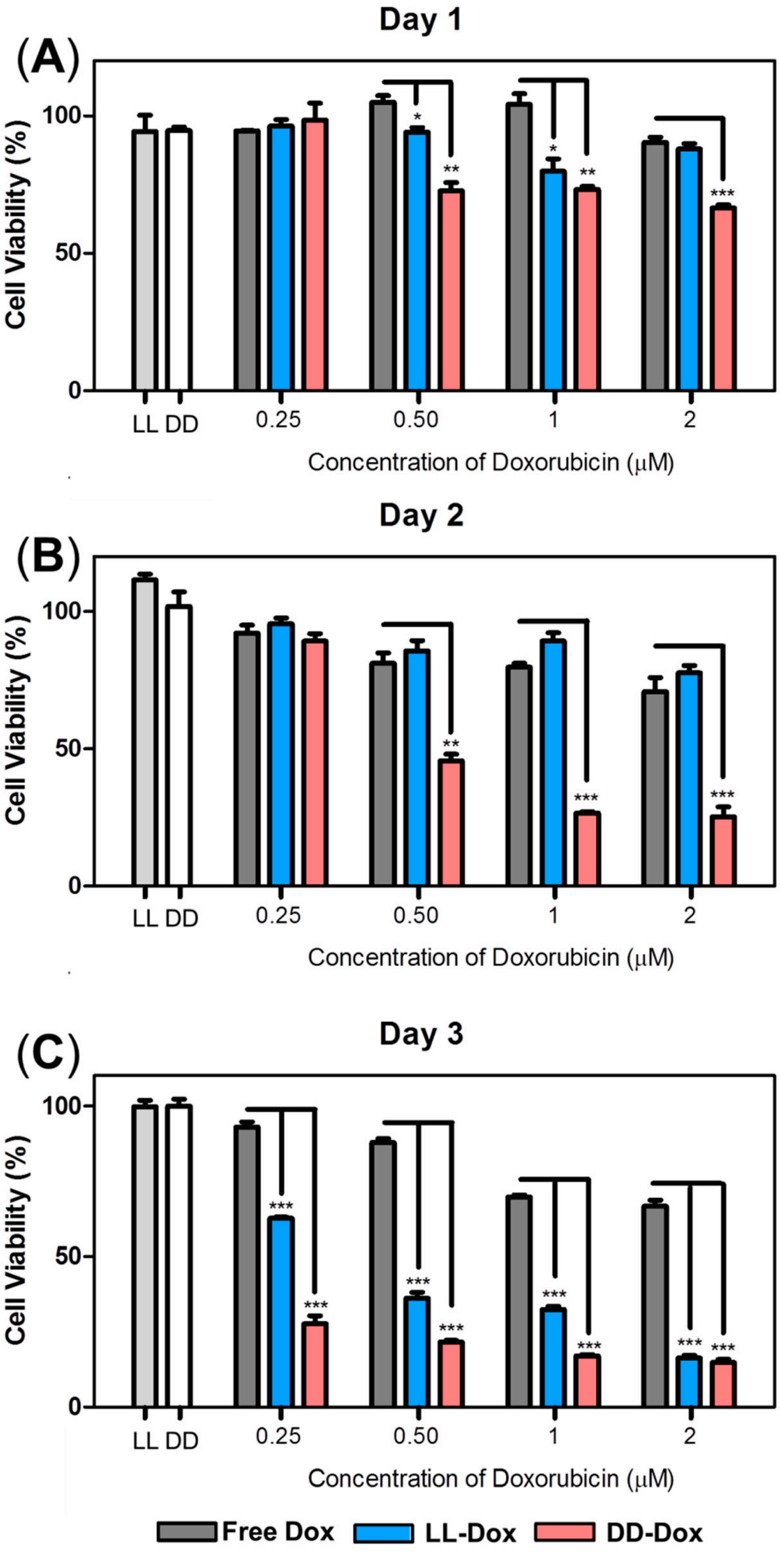

Figure S2. Anti-cancer activity of RH-$(GLFG)_3$ in HeLa cells. Viability of (A) 24-h incubation, (B) 48h-incubation, and (C) 72h-incubation. Values are reported as mean ± SEM (n=3). Statistical analyis by one-way Anova, $*p < 0.05$, $**p < 0.01$, and $***p < 0.001$ versus free-Dox.
